# MFMamba: A Mamba-Based Multi-Modal Fusion Network for Semantic Segmentation of Remote Sensing Images

**DOI:** 10.3390/s24227266

**Published:** 2024-11-13

**Authors:** Yan Wang, Li Cao, He Deng

**Affiliations:** 1 School of Electrical and Electronic Engineering, Wuhan Polytechnic University, Wuhan 430023, China; 2 School of Computer Science and Technology, Wuhan University of Science and Technology, Wuhan 430081, China; denghe@wust.edu.cn

**Keywords:** semantic segmentation, multi-modal remote sensing data, feature fusion

## Abstract

Semantic segmentation of remote sensing images is a fundamental task in computer vision, holding substantial relevance in applications such as land cover surveys, environmental protection, and urban building planning. In recent years, multi-modal fusion-based models have garnered considerable attention, exhibiting superior segmentation performance when compared with traditional single-modal techniques. Nonetheless, the majority of these multi-modal models, which rely on Convolutional Neural Networks (CNNs) or Vision Transformers (ViTs) for feature fusion, face limitations in terms of remote modeling capabilities or computational complexity. This paper presents a novel Mamba-based multi-modal fusion network called MFMamba for semantic segmentation of remote sensing images. Specifically, the network employs a dual-branch encoding structure, consisting of a CNN-based main encoder for extracting local features from high-resolution remote sensing images (HRRSIs) and of a Mamba-based auxiliary encoder for capturing global features on its corresponding digital surface model (DSM). To capitalize on the distinct attributes of the multi-modal remote sensing data from both branches, a feature fusion block (FFB) is designed to synergistically enhance and integrate the features extracted from the dual-branch structure at each stage. Extensive experiments on the Vaihingen and the Potsdam datasets have verified the effectiveness and superiority of MFMamba in semantic segmentation of remote sensing images. Compared with state-of-the-art methods, MFMamba achieves higher overall accuracy (OA) and a higher mean F1 score (mF1) and mean intersection over union (mIoU), while maintaining low computational complexity.

## 1. Introduction

Semantic segmentation of remote sensing images is a pixel-level classification task that aims to assign a specific land cover class to each pixel [1]. The derived classification information holds significant value across various fields, such as land cover [2], change detection [3], environmental protection [4], and building extraction [5]. The progression in earth observation technology has rendered the acquisition of diverse high-resolution remote sensing data more accessible [6]. This includes, but is not limited to, multi-spectral imaging (MSI), synthetic aperture radar (SAR), and light detection and ranging (LiDAR) technologies, each offering unique insights for detailed analysis and classification. Exploiting the complementary characteristics from different modalities can significantly enhance semantic segmentation performance [7]. Nonetheless, high-resolution remote sensing data are characterized by several distinct attributes, such as complex backgrounds, rich feature details, substantial intra-class variance, and relatively limited inter-class variance. Consequently, the extraction of significant features from different modal images prior to fusion remains a considerable challenge.

Traditional approaches, such as the Support Vector Machine (SVM) [8], Random Forest [9], and Conditional Random Field (CRF) [10], have been found wanting in addressing the requirements of modern high-resolution remote sensing image (HRRSI) applications due to their limitations stemming from a lack of robust abstraction and semantic feature extraction capabilities. In recent years, the predominant methods for semantic segmentation of HRRSIs have shifted towards deep learning techniques. The prevailing models based on deep learning can be broadly categorized into two main types: Convolutional Neural Networks (CNNs) and Vision Transformers (ViTs). These methods employ data-driven automation technology, allowing for autonomous learning from datasets and they generally outperform traditional approaches due to their advanced feature extraction capabilities. Nevertheless, CNNs face limitations in capturing long-range dependencies due to the inherent constraints of convolutional operations. On the other hand, ViTs excel in extracting global contextual information, though their effectiveness is contingent upon access to substantial memory and computational resources. Recently, Mamba [11], a novel state space model (SSM) originating from the natural language processing (NLP) field, has been introduced as a potential alternative for establishing long-range dependency relationships while maintaining linear computational complexity [12]. Although these deep learning-based methods have shown promise in feature extraction from single-modal images, relying solely on single-modal images for semantic segmentation limits the full utilization of available multi-modal images [13]. Compared with conventional single-modal segmentation approaches, models based on multi-modal fusion can harness target features from multiple perspectives, thereby enhancing segmentation performance [7]. However, current multi-modal fusion models based on CNNs or ViTs face challenges in capturing remote dependencies or reducing computational complexity, as well as in dealing with the incompatibility of multi-modal data.

To overcome the aforementioned challenges in semantic segmentation of remote sensing images, this paper proposes a multi-modal fusion network based on Mamba, namely MFMamba. Specifically, our MFMamba employs a dual-branch encoding structure, where the main branch extracts the features of HRRSIs utilizing a CNN-based encoder, while the auxiliary branch extracts the features of the corresponding digital surface model (DSM) based on a Mamba-based encoder. The features extracted from each stage of the two encoders are fused by an innovative feature fusion block (FFB). Subsequently, the fused features are input into the next stage of the main encoder and fed to the corresponding decoding layer as well. Finally, the segmentation result is obtained by a Transformer-based decoder.

Overall, this paper makes the following contributions:

1. A novel multi-modal fusion network based on Mamba (named as MFMamba) is proposed for semantic segmentation of remote sensing images. In this novel network, a Mamba-based auxiliary encoder is utilized to effectively capture global information from the DSM, while ensuring the network maintains low computational complexity.
2. An innovative feature fusion block (FFB) is designed to effectively fuse the features extracted from HRRSI and its corresponding DSM data, where the multi-convolutional kernel attention (MCKA) unit can further capture local details, while the efficient additive attention (EAA) unit can effectively capture long-range dependencies.
3. Extensive comparison experiments conducted on the Vaihingen dataset and the Potsdam dataset demonstrate that our proposed MFMamba has superior semantic segmentation performance and low computational complexity compared with seven state-of-the-art methods.

## 2. Related Work

### 2.1. Single-Modal Semantic Segmentation

For single-modal remote sensing images, the Fully Convolutional Network (FCN) was pioneering in its use of full convolution for pixel-level prediction [14], effectively creating a CNN architecture that addressed the semantic segmentation problem in an end-to-end manner and significantly advanced the development of semantic segmentation models. However, the sampling operation of the FCN on resolution recovery is straightforward, which can negate the impact of global contextual information. This method also encounters challenges, such as edge blurring and imprecise segmentation. To mitigate these issues, Unet was proposed as a network comprising a pyramid encoder and a symmetric decoder [15]. The encoder facilitates the extraction of multi-scale features via a progressive downsampling process, while the decoder recovers spatial resolution and contextualizes the semantic information. Following this innovation, the encoder–decoder framework has become the norm for remote sensing image segmentation networks [16]. Nevertheless, the size of the convolution kernel is limited by the convolution operation itself, preventing it from being too large. Each convolutional kernel primarily focuses on local information within its receptive field. As a result, CNN-based methods are not effective in capturing global semantic information and long-range dependencies within input images [17,18].

In order to address these challenges, the ViT was the first to introduce Transformers into computer vision tasks [19], leveraging its ability to capture long-range dependencies and yielding promising results in image classification. SegFormer further refined the architecture of the ViT to make it more suitable for semantic segmentation [20]. TransUNet employed a Transformer-based encoder and a UNet decoder for medical image segmentation [21]. The ViT excels in sequence-to-sequence modeling and significantly outperforms CNN-based models in extracting global contextual information [7]. However, the self-attention mechanism of Transformer-based models also results in a quadratic complexity with respect to input size [11], and a considerable computational load [22].

Recently, Mamba has garnered significant attention for its capability of establishing long-range dependencies while maintaining linear computational complexity [11] and has shown great potential in language understanding and vision-related applications [23], including language recognition [11], medical image segmentation [24,25], image classification [26], and 3D scene understanding [27]. Following these successes, Mamba has also demonstrated promising advancements in the domain of remote sensing imagery. One example is the novel remote sensing image classification system proposed in RSMamba [26]. In addition, RSCaMa incorporated SSM into remote sensing image change captioning (RSICC) [28], utilizing multiple CaMa layers for iterative spatial change sensing and temporal interactions. Pan-Mamba explored the application of Mamba in the field of pan-sharpening and proposed a new pan-sharpening network [29]. Samba introduced a new semantic segmentation framework for HRRSIs based on Mamba, utilizing an encoder–decoder architecture [30]. Despite the development of Mamba-based methods in single-modal remote sensing image processing, there has been no exploration into applying Mamba for semantic segmentation of multi-modal remote sensing data.

### 2.2. Multi-Modal Semantic Segmentation

In contrast to images employed in traditional computer vision tasks, HRRSIs encompass extensive geographical areas. The limited spectral characteristics of HRRSIs make low-level inter-class differences diverse, which are further complicated by the presence of shadows, noise, obstacles, geometric distortions, and building height variations [13]. Consequently, semantic segmentation relying solely on single-modal HRRSIs frequently encounters significant challenges. As the DSM encapsulates the elevation data of surface features, it can provide crucial information for the identification of highly consistent categories within an image, as well as for the clear delineation of boundaries between different categories based on elevation data. Therefore, introducing the features of the DSM can significantly improve overall segmentation accuracy [31].

To address the multi-modal fusion problem in remote sensing images, three different fusion strategies have been explored: early fusion, middle fusion, and late fusion [32]. As an early or data-level fusion approach, ResUNet-a incorporated RGB and DSM data with structural information as input into a multi-modal network [33]. Generally, early fusion requires the precise alignment of multi-modal images, yet this process frequently results in the generation of erroneous or uncorrelated features during network training. Consequently, the potential for effectively leveraging the complementary attributes of different modalities is significantly diminished [13]. In contrast, as a late or decision-level fusion method, VFuseNet employed a two-branch network to fuse RGB and DSM data after the decoding stage [34]. Similarly, the boundary detection-based semantic segmentation method proposed by Marmanis et al. also performed fusion at the final prediction stage [18]. Compared with early fusion, late fusion offers greater scalability and flexibility. However, it may suffer from insufficient cross-modal correlation [13]. Middle or feature-level fusion methods, such as FuseNet [35], employed a simple two-branch network to fuse RGB and DSM data just before the decoder stage, and cross-modal features were combined through element-wise summation and merged at various scales. However, middle fusion is only in the decoder part of the fusion, resulting in a deficiency of information interaction with the decoder. Audebert et al. extended the application of FuseNet for cross-modal fusion by incorporating residual correction in late fusion [34]. HAFNet introduced a feature-level fusion network based on a hybrid attention perception mechanism [36], while CMGFNet developed a gated fusion module to adaptively learn discriminative features and remove irrelevant information [13]. Nonetheless, these methods often fall short in effectively extracting global semantics, as they ignore long-range spatial relations [7].

Recently, Transformers have been used for multi-modal fusion [37,38] and for fusing different modalities in semantic segmentation due to their proficiency in extracting global contextual information [39]. CMFNet [31] introduced a multi-modal multi-scale fusion network based on the improved skip connection within Transformers. MFTransNet [40] presented a multi-modal semantic segmentation structure that combines CNNs with Transformers. FTransUNet [7] introduced a multi-modal semantic segmentation network that fuses shallow and deep features in a multi-level way, thereby capturing both local details and global semantics. Although multi-modal Transformers are able to capture long-range dependencies and achieve multi-scale feature fusion, their high computational complexity poses significant challenges in terms of considering model efficiency and memory footprint [12].

As Mamba has shown strong potential for establishing remote dependencies, some recent works directly use the SSM within Mamba as a module, without in-depth design for specific tasks [41]. Furthermore, the application of Mamba to multi-modal tasks has not been thoroughly investigated. Even though MambaReID [42] introduced a multi-modal fusion network based on Mamba for multi-modal object re-identification (ReID) and Sigma presented a semantic segmentation network that incorporates an attention-based Mamba fusion mechanism along with a channel-aware Mamba decoder [41], there is no Mamba-based method dedicated to semantic segmentation of multi-modal remote sensing data. Our proposed MFMamba, which leverages CNNs to extract local features and Mamba to capture global contextual information and which has feature fusion facilitated by the fusion blocks (FFBs), represents a pioneering endeavor in semantic segmentation of multi-modal remote sensing data.

## 3. Method

In this section, we provide a comprehensive overview of MFMamba, followed by detailed descriptions of its main constituent modules and, finally, a brief introduction of the used loss function is given.

### 3.1. Framework of MFMamba

**Figure 1:**
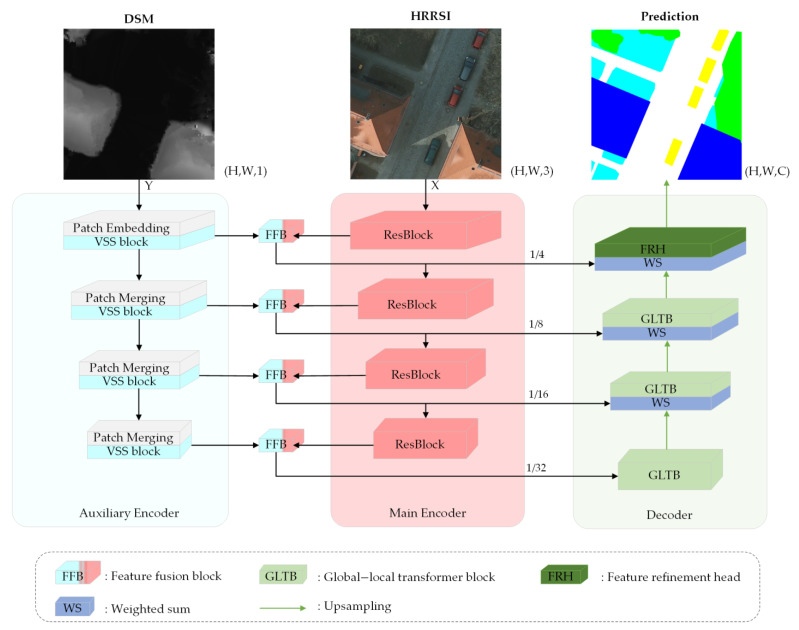
The overall architecture of our proposed MFMamba.

The framework of MFMamba is illustrated in Figure 1, which mainly consists of four parts: a Mamba-based auxiliary encoder, a CNN-based main encoder, feature fusion blocks (FFBs), and a Transformer-based decoder. Specifically, the CNN-based main encoder is employed to extract local features from the HRRSIs, while the Mamba-based auxiliary encoder is employed to extract global features from the corresponding DSM data.

Following the feature extraction at stage *i*, $i\in \left\{1,2,3,4\right\}$, an FFB is used to fuse the features extracted from both encoder branches, generating multi-scale fusion features. At each stage, these fused features are then skip-connected to the features of the corresponding layer in the Transformer-based decoder. In particular, these fused features, along with the features generated by the global–local transformer block (GLTB) from the previous layer in the decoder [43], are aggregated together to contribute to segmentation accuracy. This aggregation is selectively weighted by the weighted sum (WS) operation, aimed at learning more generalized fusion features. The formulation of the WS operation can be described as

(1) $$F_{d}^{i}=\beta \cdot F_{f}^{i}+\left(1-\beta \right)\cdot F_{g}^{i+1},$$

where $F_{d}^{i}$ represents the aggregated features at stage *i* in the decoder, $F_{f}^{i}$ denotes the fused features produced by the FFB at stage *i*, $F_{g}^{i+1}$ indicates the features generated by the GLTB at stage *i* + 1, $i\in \left\{1,2,3\right\}$, and β is the learnable weight. Finally, the aggregated features at stage 1 are processed by the feature refinement head (FRH) to generate the final prediction result.

### 3.2. Mamba-Based Auxiliary Encoder

As shown in Figure 1, the Mamba-based auxiliary encoder is structured into four successive stages. The first stage contains a patch embedding layer followed by a visual state space (VSS) block, whereas each of the remaining stages consists of a patch merging layer and a VSS block [12]. The auxiliary encoder receives the DSM data as input, which is denoted as $Y\in {\mathbb{R}}^{H\times W\times 1}$, where *H* and *W* respectively represent the height and width of the input. In the first stage, *Y* is divided into non-overlapping patches of size 2 × 2 by the patch embedding layer. The embedded image is then processed by the first VSS block. In contrast to the first stage, the remaining three stages commence with a patch merging operation that reduces the height and width of the input features while expanding the number of channels.

**Figure 2:**
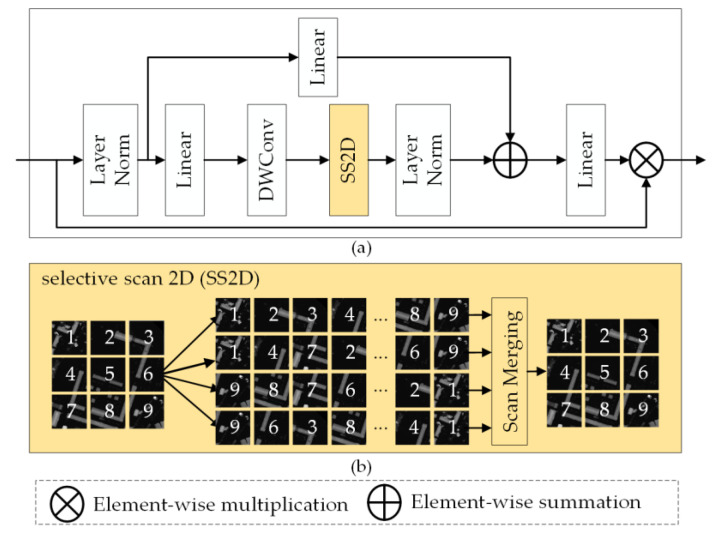
(**a**) The detailed architecture of a VSS block. (**b**) The visualization of an SS2D unit.

The VSS block, derived from VMamba [44], is the core module of the auxiliary encoder, as illustrated in Figure 2a. The input feature is processed through a series of operations, including the layer normalizations (Layer Norm), the linear projections (Linear), the depth-wise convolution (DWConv) utilized in the original Mamba, and the selective scan 2D (SS2D) unit used to model long-range spatial information from the feature. Within the SS2D unit, given an input feature map *y*, the output feature map $\bar{y}$ of the SS2D can be expressed as

(2) $$y_{s}=S6\left[scan\left(y,s\right)\right],$$

(3) $$\bar{y}=merge\left(y_{1},y_{2},y_{3},y_{4}\right),$$

where s∈{1,2,3,4} denotes four different scanning directions (left to right, right to left, top to bottom, and bottom to top) and where *scan* (·), *S*6 [·], and *merge* (·) denote the cross scan, the selective scan of the *S*6 block, and the scan merging operation, respectively. Figure 2b shows the visualization of the SS2D mechanism applied to the DSM data. The SS2D unit first unfolds the input patches into sequences along four distinct traversal paths. Each patch sequence is then independently processed through a dedicated *S*6 block [44], and its resulting sequence is subsequently reshaped and merged to form the output feature map. The detailed structure of the *S*6 block can be found in [44].

### 3.3. CNN-Based Main Encoder

The ResNet18 is utilized as the CNN-based main encoder for processing HRRSIs. Assuming that the input is represented as $X\in {\mathbb{R}}^{H\times W\times 3}$, *H* and *W* respectively represent the height and width of the input. As illustrated in Figure 1, the ResNet18 encompasses four sequential ResBlocks, each of which performs downsampling on the feature map with a scale factor of two. In order to enhance the utilization of multi-modal feature information, with the exception of the initial ResBlock, the inputs for the subsequent three ResBlocks are the fused features that have been processed through the FFBs. Moreover, skip connections are utilized by directly feeding the outputs of the FFBs into the corresponding decoder layers, which are designed to recover local details and contextual information.

### 3.4. Feature Fusion Block

**Figure 3:**
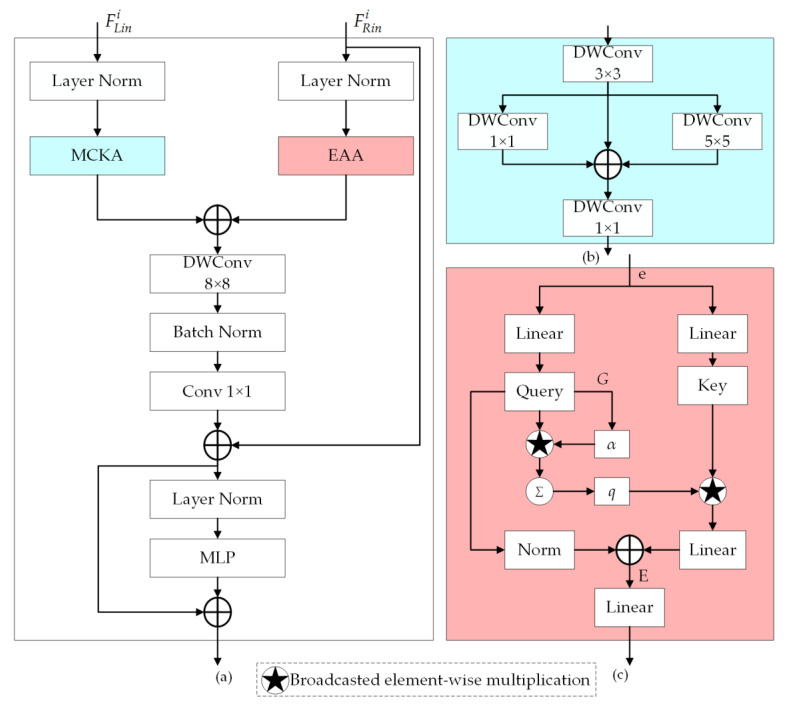
(**a**) The overall architecture of an FFB. (**b**) The structure of an MCKA unit. (**c**) The structure of an EAA unit.

The overall architecture of an FFB is illustrated in Figure 3a. At each stage, the left input $F_{Lin}^{i}$ originates from the output of the VSS block in the auxiliary encoder branch, while the right input $F_{Rin}^{i}$ is derived from the output of the ResBlock in the main encoder branch.

Prior to the fusion of the two input features, given that the left input derived from the VSS block is with long-range properties, while the right input derived through convolution operations primarily encapsulates local attributes with a limited incorporation of global information, it is necessary to employ the multi-convolutional kernel attention (MCKA) unit on the left branch in order to further capture local details and to employ the efficient additive attention (EAA) unit [45] on the right branch in order to effectively capture long-range dependencies. The first fusion of input features from both encoder branches is accomplished through the element-wise summation operation performed on the outputs from the MCKA unit and the EAA unit. In order to further extract features efficiently, the fused features are then subjected to deep separable convolution processing, where a convolution kernel of 8 × 8 is employed in the depth-wise convolution to extract spatial features, and a convolution kernel of 1 × 1 is subsequently utilized in the point-wise convolution to extract channel features. This approach enables a reduction in computation while simultaneously enhancing computation efficiency, without compromising the overall performance of the network. Considering that the DSM may exhibit varying degrees of information loss and noise, whereas the HRRSI contains crucial information that requires refinement, the input feature $F_{Rin}^{i}$ extracted from the HRRSI is once more employed for fusion with the processed features through the element-wise summation operation, thus further enhancing the detailed features. Prior to being processed by a multilayer perceptron (MLP) unit, the features—after secondary fusion—are subjected to a process of layer normalization, which makes the training process converge faster. The final fusion features are obtained by the residual connection between the outputs of the MLP unit and the features after secondary fusion.

The detailed structure of an MCKA unit is depicted in Figure 3b, which consists of a 3 × 3 convolutional layer to capture local information, followed by two parallel convolutional layers with kernel sizes of 5 and 1, respectively, enabling the capture of contextual information across various scales [46]. Finally, the local and contextual features are combined through a 1 × 1 convolutional layer to capture a wide range of contextual information without compromising the integrity of local texture features.

The detailed structure of an EAA unit is shown in Figure 3c. The conventional attention mechanism in natural language processing (NLP) encodes a relevance score for the contextual information of the input sequence based on the interaction among three attention components: query, key, and value. In contrast, the EAA mechanism eliminates key–value interactions while maintaining performance and focuses on effectively encoding query–key interactions through the incorporation of a linear projection layer. The input embedding matrix e is transformed into Query and Key, where Query, Key$\in {\mathbb{R}}^{n\times d}$, *n* is the token length, and *d* is the dimensions of the embedding vector. Next, the Query is multiplied by the learnable parameter vector $G\in {\mathbb{R}}^{d}$ to learn the attention weights of the query, producing the global attention query vector $\alpha \in {\mathbb{R}}^{n}$ as follows:

(4) $$\alpha =\frac{\mathrm{Query}\cdot G}{\sqrt{d}}.$$

Then, the Query is pooled based on the learned attention weights, resulting in a single global query vector $q\in {\mathbb{R}}^{d}$ as follows:

(5) $$q=\sum_{i=1}^{n}\alpha_{i}*{\mathrm{Query}}_{i}.$$

Here, ∗ denotes the broadcasted element-wise multiplication operation. Finally, the output of the EAA unit E can be described as:

(6) $$E=\hat{Q}+T(\mathrm{Key}*q),$$

where $\hat{Q}$ denotes to the normalized Query, T denotes to the linear transformation.

### 3.5. Transformer-Based Decoder

**Figure 4:**
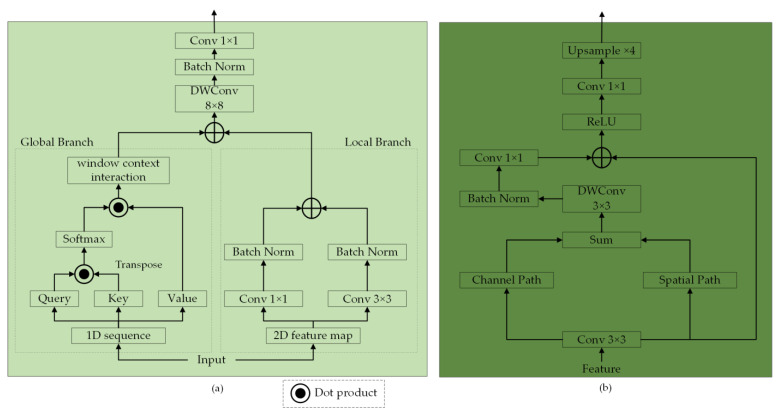
(**a**) The structure of a GLTB. (**b**) The structure of an FRH.

The challenge of multi-scale issues in remote sensing images complicates target localization and recognition. Conventional decoders often struggle to accurately recover image details due to the absence of global semantic information [43]. The decoder in UNetFormer effectively addresses this by capturing global and local contextual information across multiple scales [43]. As depicted in Figure 1, the Transformer-based decoder mainly consists of three GLTBs and an FRH. The detailed structure of a GLTB is shown in Figure 4a, which contains two parallel branches to extract global and local context, respectively. The local branch adopts two parallel convolutional layers with kernel sizes of 3 and 1 to extract local context, while the global branch relies on the multi-head self-attention of the window to capture global context. Because the shallow features generated from the FFB at stage 1 retain rich spatial details yet fall short in semantic content and because the features processed by the GLTB at stage 2 provide precise semantic information but with low spatial resolution, the FRH is used at stage 1 to narrow the semantic gap between the two features, thereby further improving overall accuracy. As illustrated in Figure 4b, the detailed structure of an FRH features two parallel pathways that are designed to enhance the channel-wise and spatial-wise feature representations, and the attentional features generated by the two pathways are further fused by summation operations. Subsequent to this fusion, convolution and upsampling operations are employed to yield the final segmentation map. Detailed descriptions of the GLTB and the FRH can be found in UNetFormer [43].

### 3.6. Loss Function

Based on the multi-head design in the Transformer-based decoder, the loss function applied in this paper is a combination of the Cross-entropy loss and the Dice loss.

The Cross-entropy loss is expressed as follows:

(7) $$L_{\mathrm{ce}}=-\frac{1}{N}\sum_{n=1}^{N}\sum_{k=1}^{K}y_{k}^{\left(n\right)}\log{\hat{y}}_{k}^{\left(n\right)},$$

where *N* is the number of samples, *K* represents the number of categories, $y_{k}^{\left(n\right)}$ represents the one-hot encoding of the true semantic labels, and ${\hat{y}}_{k}^{\left(n\right)}$ is the confidence of sample *n* belonging to the category *k*.

The Dice loss is expressed as follows:

(8) $$L_{\mathrm{dice}}=1-\frac{2}{N}\sum_{n=1}^{N}\sum_{k=1}^{K}\frac{y_{k}^{\left(n\right)}{\hat{y}}_{k}^{\left(n\right)}}{y_{k}^{\left(n\right)}+{\hat{y}}_{k}^{\left(n\right)}}.$$

The total loss function is expressed as:

(9) $$L_{\mathrm{total}}=L_{\mathrm{ce}}+L_{\mathrm{dice}}+\gamma \times L_{\mathrm{GLTB}},$$

where $L_{\mathrm{GLTB}}$ is the loss of operations on three GLTB blocks. The detailed description of $L_{\mathrm{GLTB}}$ can be found in UNetFormer [43]. The symbol γ is the cofactor and is set to 0.4 by default.

## 4. Experiments and Results

### 4.1. Datasets

The Vaihingen dataset consists of 16 very high-resolution true orthophotos (TOPs), each with an average size of 2500 × 2000 pixels. Every orthophoto has three channels, namely near-infrared, red, and green (NIRRG), along with a DSM with a 9 cm ground sampling distance (GSD). The dataset consists of five foreground classes, namely *Impervious surface* (Imp.), *Building* (Bui.), *Low vegetation* (Low.), *Tree* (Tre.), and *Car*, as well as one background class (*Clutter*). In our experiments, we utilized TOP image tiles and complete images. The 16 orthophoto images and their corresponding DSM data were divided into a training set containing 12 patches and a test set containing 4 patches. The training set comprises images identified by the indices 1, 3, 23, 26, 7, 11, 13, 28, 17, 32, 34, and 37. The test set consists of images identified by the indices 5, 21, 15, and 30.

**Figure 5:**
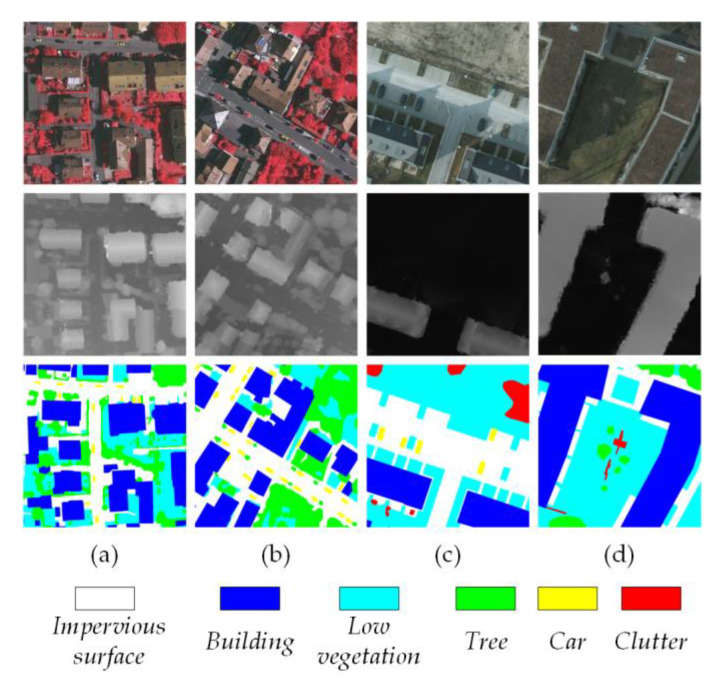
Samples (**a**,**b**) are 256 × 256 from Vaihingen and (**c**,**d**) are 256 × 256 from Potsdam. The first row shows the orthophotos with three channels (NIRRG for Vaihingen and RGB for Potsdam). The second and third rows show the corresponding depth information and semantic labels in pixel-wise mapping.

The Potsdam dataset is composed of 24 very high-resolution TOPs, each with a size of 6000 × 6000 pixels, involving the same category information as the Vaihingen dataset. Distinct from the Vaihingen dataset, it provides four multi-spectral channels, including infrared, red, green, and blue (IRRGB), along with a normalized DSM at 5 cm GSD. In our experiments, we selected to use their RGB composites and the corresponding DSM data. The 24 orthophoto images and their corresponding DSM data were divided into a training set containing 18 patches and a test set containing 6 patches. The training set includes images identified by the following indices: 6_10, 7_10, 2_12, 3_11, 2_10, 7_8, 5_10, 3_12, 5_12, 7_11, 7_9, 6_9, 7_7, 4_12, 6_8, 6_12, 6_7, and 4_11. The test set is composed of images with the indices 2_11, 3_10, 4_10, 5_11, 6_11, and 7_12. Figure 5 presents a selection of data samples from the Vaihingen and the Potsdam datasets.

### 4.2. Evaluation Metrics and Experimental Setup

In order to quantitatively evaluate segmentation performance, we utilized overall accuracy (OA) and the mean F1 score (mF1) and mean intersection over union (mIoU) as our evaluation metrics. Based on the accumulated confusion matrix, the calculations for OA and the mF1 and mIoU can be expressed as follows:

(10) $$\mathrm{OA}=\frac{\sum_{k=1}^{N}TP_{k}+TN_{k}}{\sum_{k=1}^{N}TP_{k}+FP_{k}+TN_{k}+FN_{k}},$$

(11) $$Q_{p}=\frac{1}{N}\overset{N}{\underset{k=1}{\sum }}\frac{TP_{k}}{TP_{k}+FP_{k}},$$

(12) $$Q_{r}=\frac{1}{N}\overset{N}{\underset{k=1}{\sum }}\frac{TP_{k}}{TP_{k}+FN_{k}},$$

(13) $$F1=2\times \frac{Q_{p}\times Q_{r}}{Q_{p}+Q_{r}},$$

(14) $$\mathrm{mIoU}=\frac{1}{N}\overset{N}{\underset{k=1}{\sum }}\frac{TP_{k}}{TP_{k}+FP_{k}+FN_{k}},$$

where $TP_{k}$, $FP_{k}$, $TN_{k}$, and $FN_{k}$ denote true positives, true negatives, false positives, and false negatives, respectively, for objects indexed as class k. Specifically, we incorporated *Clutter* into the evaluation of OA and calculated the mF1 and mIoU values for five foreground classes.

All the experiments were conducted within the PyTorch framework on an NVIDIA A40 GPU with 48 GB RAM. During the training process, the images were randomly cropped into 256 × 256 patches, and data augmentation techniques were adopted such as random vertical flip, random horizontal flip, and random rotations. The training epoch was set as 50. The models were trained using the stochastic gradient descent (SGD) algorithm with the following parameters: a learning rate of 0.01, a momentum of 0.9, and a decay coefficient of 0.0005. In order to determine the optimal settings of hyperparameters for our proposed MFMamba, a series of experiments were conducted to adjust and optimize various hyperparameter settings. The quantitative results of our model with different batch sizes and learning rate schedulers are illustrated in Table 1. It can be seen that the best results are obtained by setting the batch size to 32 with a learning rate scheduler set to decay at the 10th, 20th, and 30th training epochs.

### 4.3. Experimental Results

We have adopted seven state-of-the-art methods for performance comparison, which include two CNN-based approaches named as ABCNet [47] and MAResU-Net [48], four Transformer-based techniques named as CMFNet [31], TransUNet [21], UNetFormer [43], and CMTFNet [49], as well as one Mamba-based method named as RS3Mamba [12].

#### 4.3.1. Comparison Results on the Vaihingen Dataset

As can be seen from Table 2, our proposed MFMamba attains the highest scores for OA and the mF1 and mIoU on the Vaihingen dataset. Notably, our MFMamba exhibits significant enhancements in terms of OA and the mIoU when compared with the baseline RS3Mamba, with increments of 0.51% and 0.57%, respectively. This validates the efficacy of our proposed dual-branch encoding architecture for extracting multi-modal features. In comparison with the existing state-of-the-art methods, MFMamba outperforms in segmenting four classes: *Impervious surface*, *Building*, *Tree*, and *Car*. In particular, the F1 score for *Impervious surface* is increased by 3.11% and for *Building* by 2.66%, outperforming the CMFNet.

**Figure 6:**
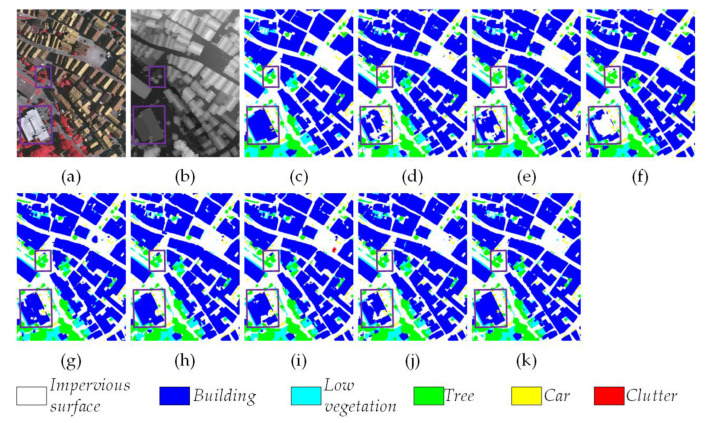
Visualization of the segmentation results from different methods on the Vaihingen dataset. (**a**) NIRRG images, (**b**) DSM, (**c**) Ground Truth, (**d**) CMFNet, (**e**) ABCNet, (**f**) TransUNet, (**g**) UNetFormer, (**h**) MAResU-Net, (**i**) CMTFNet, (**j**) RS3Mamba, and (**k**) the proposed MFMamba. Two purple boxes are added to each subfigure to highlight the differences.

Figure 6 illustrates a visualization of the results achieved by all eight methods, which also serves to highlight the effectiveness of our MFMamba. It can be observed that HRRSIs possess more intricate backgrounds and a greater abundance of intricate details in comparison with natural images. As illustrated in Figure 6, our proposed MFMamba is capable of more accurately classifying *Building* and *Impervious surface*. In addition, the classification of *Tree* and *Car* is more closely aligned with the Ground Truth. In order to highlight the segmentation results, two purple boxes are added to each subfigure in Figure 6. We can see that in the lower left box, our MFMamba splits *Building* arranged in it relatively completely. In the upper box, our MFMamba effectively identifies *Tree* around *Building*, providing a more tidy and complete segmentation of *Tree*.

#### 4.3.2. Comparison Results on the Potsdam Dataset

The experiments conducted on the Potsdam dataset also yielded results similar to those obtained from the Vaihingen dataset. As illustrated in Table 3, the segmentation performance for *Impervious surface*, *Building*, *Low vegetation*, and *Tree* were 93.31%/87.46%, 97.81%/95.75%, 86.76%/76.62%, and 87.19%/77.28%, respectively, representing an improvement of 0.11%/0.19%, 0.49%/0.96%, 0.69%/1.08%, and 0.59%/0.91% in F1 and IoU, respectively, compared with the baseline RS3Mamba. When compared with existing state-of-the-art methods, MFMamba outperforms in segmenting three classes: *Impervious surface*, *Building*, and *Low vegetation*.

**Figure 7:**
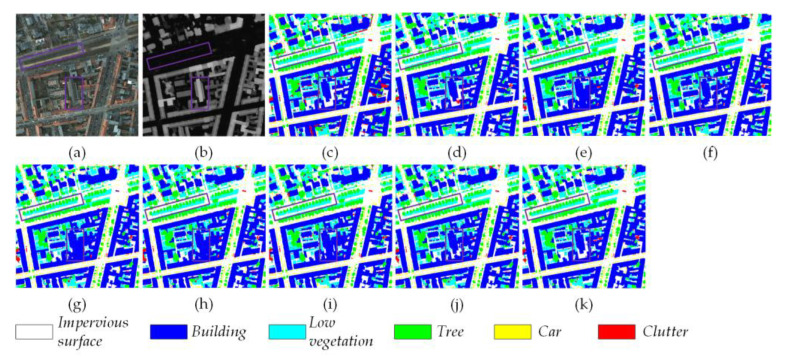
Visualization of the segmentation results from different methods on the Potsdam dataset. (**a**) RGB images, (**b**) DSM, (**c**) Ground Truth, (**d**) CMFNet, (**e**) ABCNet, (**f**) TransUNet, (**g**) UNetFormer, (**h**) MAResU-Net, (**i**) CMTFNet, (**j**) RS3Mamba, and (**k**) the proposed MFMamba. Two purple boxes are added to each subfigure to highlight the differences.

Figure 7 shows a visualization example from the Potsdam dataset for all eight methods under consideration. It can be observed by the highlighted two purple boxes in each subfigure that, in the upper left box, our proposed MFMamba more accurately segments the *Low vegetation* in *Tree* region and that, in the lower box, our MFMamba divides *Building* more completely and contains less *Clutter*. Clearly, our MFMamba is capable of effectively detecting intricate edges and generating smoother results. Our approach produces more comprehensive and cohesive results, reducing the number of independent points.

#### 4.3.3. Analysis of Computational Complexity

We employed floating-point operations (FLOPs) and the number of model parameters as our evaluation criteria to evaluate the computational complexity of our proposed MFMamba. FLOPs can be used to assess the temporal complexity of a deep learning-based model, whereas the number of model parameters serves to quantify the size of the model. An ideal model would exhibit a reduction in the numbers of floating-point operations and model parameters while preserving a high standard of processing performance.

The first two columns in Table 4 present the numbers of floating-point operations and model parameters of all methods considered in this paper, and the last column shows their corresponding processing performance on the Vaihingen dataset. Table 4 shows that, although UNetFormer has the fewest FLOPs and model parameters, its mIoU score is much lower than our model. Our MFMamba significantly reduces FLOPs and requires fewer model parameters while maintaining a higher mIoU score, in comparison with the multi-modal fusion methods like TransUNet and CMFNet. This efficiency stems from using Mamba as an auxiliary branch in the encoder, which is a less resource-intensive method than using a Transformer. Compared with single-modal segmentation methods, the computational complexity of our model is slightly higher due to the introduction of multi-modal data, but our method has better segmentation performance. Furthermore, our MFMamba provides a clear improvement in segmentation performance with a slight increase in the number of model parameters compared with the baseline RS3Mamba.

### 4.4. Ablation Study

To verify the effectiveness of introducing the DSM data, we conducted ablation experiments on the Vaihingen dataset and the Potsdam dataset by configuring the inputs as HRRSIs only and as HRRSIs combined with the DSM data, respectively. As shown in Table 5, the proposed MFMamba can improve the accuracy of most categories on both datasets by effectively utilizing the additional DSM data. In particular, there is a significant improvement for segmenting *Building*, *Tree*, and *Impervious surface*, since ground objects in these three categories typically have distinct height characteristics. In addition, as cars are usually located on roads, it is also helpful to identify the car–road boundary by the height of the road. Because the elevation characteristics of *Low vegetation* are very similar to *Impervious surface* and *Tree*, and *Low vegetation* is often occluded due to its location adjacent to Tree, our proposed MFMamba encounters challenges in improving the accuracy of *Low vegetation*.

The effectiveness of the proposed FFB was confirmed via ablation studies on the Vaihingen dataset. These studies preserved the FFB’s architecture but altered it to incorporate just a single attention, as presented in Table 6. The results in Table 6 indicate that both the EAA and MCKA units are essential components for the FFB of the proposed MFMamba to achieve better segmentation performance.

## 5. Conclusions

In this paper, we proposed a novel Mamba-based multi-modal fusion network called MFMamba, which is the first bold attempt of Mamba applied in semantic segmentation of multi-modal remote sensing data. MFMamba integrates a dual-branch encoder for feature extraction from multi-modal remote sensing data, where the CNN-based main encoder is utilized to extract the features of HRRSIs and the Mamba-based auxiliary encoder is utilized to extract the DSM features, respectively. In order to better fuse the global and local features, a new feature fusion block (FFB) is designed to fuse the features extracted from the dual-branch encoder at each stage and further generate multi-scale fusion features. Compared with the methods that use CNNs or ViTs, MFMamba provides a new idea for semantic segmentation of multi-modal remote sensing data. Comprehensive experiments conducted on two public datasets, the Vaihingen and the Potsdam, demonstrate that our proposed MFMamba outperforms other seven state-of the-art methods in terms of semantic segmentation performance with low computational complexity. Further research on real-world noise data and model robustness will be carried out to continuously improve the performance of our proposed model.

## Figures and Tables

**Table 1:** Quantitative results of our proposed MFMamba with different batch sizes and learning rate schedulers on the Vaihingen dataset (%). The best results are highlighted in red.

Batch Size	Learning Rate Scheduler	OA	mF1	mIoU
16	[25, 35, 45]	91.60	90.30	82.74
24	[25, 35, 45]	91.74	90.37	82.90
28	[25, 35, 45]	91.50	90.36	82.85
30	[25, 35, 45]	91.67	90.38	82.91
36	[25, 35, 45]	91.38	90.22	82.58
48	[25, 35, 45]	91.47	90.35	82.83
32	[25, 35, 45]	91.64	90.38	82.93
32	[15, 25, 35]	91.71	90.45	83.01
32	[10, 20, 30]	91.81	90.52	83.13

**Table 2:** Results of comparison with other methods on the Vaihingen dataset (%). The best and second-best results are respectively marked in red and blue colors.

Method	Backbone	F1/IoU					OA	mF1	mIoU
		Imp.	Bui.	Low.	Tre.	Car			
CMFNet [31]	VGG-16	90.11/81.99	94.51/89.60	77.72/63.56	90.09/81.97	86.52/76.24	89.38	87.79	78.67
ABCNet [47]	ResNet-18	92.08/85.32	95.96/92.24	79.87/66.49	90.38/82.45	85.61/74.84	90.79	88.78	80.27
TransUNet [21]	R50-ViT-B	92.21/85.54	96.10/92.48	80.79/67.77	90.87/83.27	89.60/81.16	91.21	89.91	82.04
UNetFormer [43]	ResNet-18	92.23/85.58	96.34/92.93	80.74/67.70	91.04/83.55	90.37/82.43	91.29	90.14	82.44
MAResU-Net [48]	ResNet-34	92.66/86.33	96.84/93.87	80.57/67.47	90.84/83.22	89.93/81.71	91.50	90.17	82.51
CMTFNet [49]	ResNet-50	92.68/86.37	96.71/93.63	80.47/67.33	90.78/83.11	90.22/82.18	91.42	90.17	82.52
RS3Mamba [12]	R18-Mamba-T	92.69/86.38	96.67/93.55	80.54/67.42	90.59/82.79	90.49/82.64	91.30	90.20	82.56
MFMamba (Ours)	R18-Mamba-T	93.22/87.31	97.17/94.50	80.63/67.54	91.05/83.58	90.53/82.70	91.81	90.52	83.13

**Table 3:** Results of quantitative comparison on the Potsdam dataset (%). The best and second-best results are respectively marked in red and blue colors.

Method	Backbone	F1/IoU					OA	mF1	mIoU
		Imp.	Bui.	Low.	Tre.	Car			
CMFNet [31]	VGG-16	93.09/87.07	96.90/93.99	85.88/75.26	86.52/76.25	96.17/92.62	90.72	91.71	85.04
ABCNet [47]	ResNet-18	92.90/86.74	96.99/94.16	86.11/75.62	87.02/77.02	96.31/92.88	90.82	91.87	85.28
TransUNet [21]	R50-ViT-B	93.08/87.06	96.88/93.94	86.74/76.59	87.66/78.03	96.40/93.05	91.03	92.15	85.73
UNetFormer [43]	ResNet-18	93.02/86.95	97.14/94.43	86.21/75.76	86.93/76.88	96.79/93.78	90.89	92.02	85.56
MAResU-Net [48]	ResNet-34	93.15/87.17	97.21/94.57	86.73/76.57	87.14/77.21	96.67/93.56	91.05	92.18	85.82
CMTFNet [49]	ResNet-50	93.08/87.06	97.30/94.73	86.32/75.94	87.13/77.20	96.89/93.97	90.97	92.15	85.78
RS3Mamba [12]	R18-Mamba-T	93.20/87.27	97.32/94.79	86.07/75.54	86.60/76.37	96.74/93.68	90.92	91.99	85.53
MFMamba (Ours)	R18-Mamba-T	93.31/87.46	97.81/95.75	86.76/76.62	87.19/77.28	96.63/93.48	91.38	92.34	86.12

**Table 4:** Comparison results of computational complexity and the mIoU on the Vaihingen dataset. The best and second-best results are respectively marked in red and blue colors.

Method	FLOPs (G)	Parameter (M)	mIoU (%)
CMFNet [31]	255.28	104.07	78.67
ABCNet [47]	12.58	13.67	80.27
TransUNet [21]	123.49	105.32	82.04
UNetFormer [43]	9.45	11.69	82.44
MAResU-Net [48]	23.08	26.28	82.51
CMTFNet [49]	28.68	30.07	82.52
RS3Mamba [12]	31.65	43.32	82.56
MFMamba (Ours)	30.59	62.43	83.13

**Table 5:** Ablation study of introducing DSM data on the Vaihingen and the Potsdam datasets.

Dataset	Bands	Class OA(%)				
		Imp.	Bui.	Low.	Tre.	Car
Vaihingen	NIRRG	91.58	97.01	80.08	91.24	87.86
	NIRRG + DSM	92.18 (+0.60)	97.91 (+0.90)	79.67 (−0.41)	92.08 (+0.84)	89.43 (+1.57)
Potsdam	RGB	92.65	97.94	88.60	86.40	96.69
	RGB + DSM	93.06 (+0.41)	98.29 (+0.35)	88.64 (+0.04)	87.19 (+0.79)	96.31 (−0.38)

**Table 6:** Ablation study of the proposed FFBs on the Vaihingen dataset.

MCKA	EAA	OA (%)	mF1 (%)	mIoU (%)
√		91.68	90.45	82.98
	√	91.50	90.22	82.60
√	√	91.81	90.52	83.13

## Data Availability

The code can be obtained from https://github.com/YanWang-WHPU/MFMamba (accessed on 1 October 2024). The Vaihingen and the Potsdam datasets can be obtained from https://www.isprs.org/education/benchmarks/UrbanSemLab/default.aspx (accessed on 1 October 2024).
